# Diagnosing acute appendicitis in children with neutrophil–lymphocyte ratio: a cross-sectional study

**DOI:** 10.3389/fped.2025.1656254

**Published:** 2025-11-27

**Authors:** Mohamed Mahmoud Shalaby, Ahmed Alawi, Bayan Mohammed Fatani, Ameen Alsaggaf, Mohammed Awad

**Affiliations:** 1 Pediatric Surgery Department, Faculty of Medicine, Tanta University, Tanta, Egypt; 2 Pediatric Surgery Department, King Fahd Armed Forces Hospital (KFAFH), Jeddah, Saudi Arabia; 3 Pediatric Surgery Department, King Faisal Specialist Hospital and Research Center, Jeddah, Saudi Arabia

**Keywords:** acute appendicitis, complicated, pediatric, neutrophil-to-lymphocyte ratio, white blood cell count

## Abstract

**Background:**

Diagnosing acute appendicitis (AA) in children can be challenging due to atypical symptoms and the difficulty in obtaining a comprehensive history. This study aimed to evaluate the value of the neutrophil-to-lymphocyte ratio (NLR) in diagnosing AA in the pediatric population.

**Methods:**

This retrospective cross-sectional study involved 200 pediatric patients aged between 0 and 14 years of both sexes, who underwent appendectomy for suspected acute appendicitis. Data of patients were retrieved from the hospital's patient administration system database. We categorized patients into three surgical groups: Group 1, appendectomy + normal appendix (*n* = 32); Group 2, appendectomy + acute appendicitis (AA) (*n* = 133); and Group 3, appendectomy + perforated appendicitis (*n* = 35).

**Results:**

White blood cell (WBC) count, neutrophil count, and NLR were significantly higher in the acute appendicitis and complicated appendicitis groups than those in the normal appendix group and in the complicated appendicitis group than those in the acute appendicitis group. In multivariate regression, WBC count and NLR were associated with AA (*P* ≤ 0.001). NLR was associated with complicated appendicitis (*P* = 0.012). It was also significantly associated with AA and complicated appendicitis at cutoff values >1.7 and >3.5, respectively, with 74% and 68.57% sensitivity and 69% and 56.98% specificity.

**Conclusions:**

NLR is a potentially useful adjunctive and cost-effective diagnostic marker for AA and complicated appendicitis in the pediatric population.

## Introduction

Acute appendicitis (AA) is one of the most common causes of abdominal pain in children that necessitates emergency surgery (1). Children often exhibit symptoms for an extended period and have a higher rate of perforation (31.8%–45.8%), which may be more pronounced in the general pediatric population (0–14 years) (2).

The diagnosis of AA in children under the age of five can be particularly challenging due to atypical symptoms and difficulties in obtaining a comprehensive medical history (3).

Timely diagnosis is crucial in AA, as delays can lead to an increased risk of perforation and subsequent complications (4). Despite the use of clinical assessments and imaging techniques such as ultrasound (US) and computed tomography (CT), there is a need for clear decision aids to support early AA detection (5).

Most patients have uncomplicated appendicitis, while those admitted with complicated appendicitis have higher morbidity rates and poorer surgical outcomes (6). The utility of conventional inflammatory biomarkers, including the white blood cell (WBC) count and the neutrophil-to-lymphocyte ratio (NLR) from routine complete blood count (CBC) tests, has been investigated in the diagnosis of AA (7).

NLR has recently gained attention as a potential diagnostic marker for AA due to its simplicity, cost-effectiveness, and ease of calculation from CBC tests. It reflects the balance between neutrophil-mediated inflammation and lymphocyte response, which may be altered in inflammatory conditions such as appendicitis (8).

To date, no study has been conducted in Jeddah city to examine the relationship between these biomarkers and AA. Thus, this study aimed to evaluate the diagnostic value of NLR in AA in the pediatric population.

### Patients and methods

This retrospective cross-sectional study involved 200 patients who underwent appendectomy for suspected acute appendicitis aged between 0 and 14 years of both sexes. This retrospective cohort study was conducted at a single center, King Fahd Armed Forces Hospital (KFAFH), Jeddah, Saudi Arabia. Data between January 2018 and January 2024 were collected following ethical approval from the KFAFH ethical committee in Jeddah, Saudi Arabia (approval code ID: 481191). Patients with large missing data were excluded.

### Retrospective data collection

This study was conducted using a retrospective design. Data were collected from institutional medical records over the defined study period. Eligible patients were identified by reviewing surgical logs, admission records, and operative reports. Demographic variables, type of presentation, presence and type of clinical picture, operative details, and outcomes were extracted. Only patients with complete documentation of both surgical findings and clinical evaluation were included in the analysis. Cases with missing or incomplete charts were excluded.

### Sample size determination

This was a retrospective, single-center study, and a convenience sample of all eligible patients during the study period was used. A *post hoc* power analysis was conducted based on the final cohort size. Cases were included if complete demographic, operative, and follow-up data were available; patients with missing or incomplete records were excluded. The sample size was determined by the availability of complete records. Inclusion/exclusion criteria shaped the final cohort. Thus, the final sample size reflects the total number of eligible patients meeting these criteria rather than a predetermined statistical target.

The diagnosis of AA was based on clinical presentation, physical examination, laboratory results, and/or abdominal US. In this study, the gold standard diagnostic method was considered to be the intraoperative and histopathological findings.

We have categorized patients into three surgical groups:

- Group 1: appendectomy + normal appendix—patients with a macroscopically and histopathologically normal appendix upon surgery (*n* = 32).
- Group 2: appendectomy + acute appendicitis (AA)—patients with intraoperative findings of inflamed, non-perforated acute appendicitis (*n* = 133).
- Group 3: appendectomy + perforated appendicitis—patients with intraoperative evidence of appendiceal perforation or gangrene (*n* = 35).

Data on demographics, laboratory investigations (WBC, neutrophil, and lymphocyte count, NLR, and sodium level), histopathological results, diagnosis, treatment, antibiotics taken, and other medications were extracted from the hospital's patient administration system database.

### Statistical analysis

Statistical analysis was performed using SPSS v27 (IBM©, Chicago, IL, USA). The Shapiro–Wilk test and histograms were employed to evaluate the normality of the data distribution. Quantitative parametric data were presented as median [interquartile range (IQR)] and were analyzed using the Kruskal–Wallis test with a Dunn's *post hoc* test with Bonferroni correction. Qualitative variables were presented as frequency and percentage (%) and were analyzed using the chi-squared test. The overall diagnostic performance of each test was assessed using ROC curve analysis. Multivariate regression was also utilized to estimate the relationship between a dependent variable and multiple independent variables. A two-tailed *P*-value of <0.05 was considered statistically significant.

## Results

Age and sex did not show statistical significance among the three main groups (Table 1).

**Table 1:** Demographic data of the studied groups.

		Normal appendix group (*n* = 32)	Acute appendicitis group (*n* = 133)	Complicated appendicitis group (*n* = 35)	*P*
Age (years)		9.7 (4–12)	9.3 (4.5–11.5)	9.7 (4–12)	0.095
Sex	Male	15 (46.8%)	72 (54.13%)	14 (40%)	0.135
	Female	17 (53.2%)	61 (45.87%)	21 (60%)	

Data are presented as median [interquartile range (IQR)] (%).

WBC and neutrophil counts and NLR were significantly higher in the acute appendicitis and complicated appendicitis groups than those in the normal appendix group and in the complicated appendicitis group than those in the acute appendicitis group (*P* < 0.05). Lymphocytes were negligibly different among the three groups. Sodium level was significantly higher in the normal appendix group than that in the acute appendicitis and complicated appendicitis groups (*P* < 0.001) and was negligibly different between the acute appendicitis and complicated appendicitis groups (Table 2).

**Table 2:** Laboratory investigation of the studied groups.

Parameter	Normal appendix (*n* = 32)	Acute appendicitis (*n* = 133)	Complicated appendicitis (*n* = 35)	*P*-value	*Post hoc* summary
WBC (×10^3^/µL)	8 (7–10.5)	13 (7.1–18.9)	14.4 (6.0–22.8)	<0.001	P1 < 0.001, P2 < 0.001, P3 = 0.003
Neutrophil (×10^3^/µL)	4.9 (2.7–7.0)	9.8 (4.1–15.5)	11.2 (3.8–18.6)	<0.001	P1 < 0.001, P2 < 0.001, P3 = 0.002
Lymphocyte (×10^3^/µL)	2.7 (1.2–4.2)	2.4 (−0.9–5.7)	2.2 (−1.2 to –5.7)	0.086	
Neutrophil-to-lymphocyte ratio	1.5 (1.1–1.9)	5.3 (1.6–8.9)	8.2 (2.2–14.1)	<0.001	P1 < 0.001, P2 < 0.001, P3 < 0.001
Sodium (mEq/L)	140.4 (136.4–144.4)	138.1 (134.6–141.6)	138.0 (135.4–140.6)	<0.001	P1 < 0.001, P2 < 0.001, P3 = 0.985

Data are presented as median [interquartile range (IQR)]. P1: *P* between the normal appendix and acute appendicitis groups. P2: *P* between the normal appendix and complicated appendicitis groups. P3: *P* between the acute appendicitis and complicated appendicitis groups.

The way of confirming diagnosis did not show statistical significance between the acute appendicitis and complicated appendicitis groups. Antibiotics and treatment were significantly different between the acute appendicitis and complicated appendicitis groups (Table 3).

**Table 3:** Diagnosis, treatment, and antibiotics of the studied groups.

		Normal appendix group (*n* = 32)	Acute appendicitis group (*n* = 133)	Complicated appendicitis group (*n* = 35)	*P*
Way of confirming diagnosis	Clinical	17 (53.12%)	41 (33.33%)	8 (22.86%)	0.097
	US	15 (46.87)	77 (58.18%)	20 (57.14%)	
	CT	0	15 (8.48%)	7 (20%)	
Treatment	Simple appendectomy	32 (100%)	133 (100%)	0 (0%)	**<0.001**
	Complicated perforated appendectomy	0 (0%)	0 (0%)	22 (62.86%)	
	Complicated abscessed appendicitis	0 (0%)	0 (0%)	6 (17.14%)	
	Recurrent attack after previous antibiotic management	0 (0%)	0 (0%)	7 (20%)	
Antibiotics	Piperacillin tazobactam	22 (68.75%)	107 (80.45%)	23 (65.71%)	**0.006**
	Cefuroxime	3 (9.37%)	20 (15.03%)	11 (31.43%)	
	Metronidazole	0 (0%)	20 (15.03%)	11 (31.43%)	
	Amoxicillin	0 (0%)	2 (1.50%)	1 (2.86%)	
	Vancomycin	5 (15.62%)	3 (2.25%)	0 (0%)	
	Trimethoprim/sulfamethoxazole	0 (0%)	1 (0.75%)	0 (0%)	
	Ciprofloxacin	0 (0%)	1 (0.75%)	0 (0%)	
	Gentamicin	0 (0%)	0 (0%)	4 (11.43%)	
	Amoxicillin/clavulanic acid	2 (6.25%)	2 (1.5%)	1 (2.86%)	
Histopathology	No significant pathology abnormality	31 (96.87%)	0 (0%)	0 (0%)	1
	Normal appendix with fecal impaction	1 (3.12%)	0 (0%)	0 (0%)	1
	Acute simple appendicitis	0 (0%)	129 (96.99%)	1 (4.35%)	**<0.001**
	Acute suppurative appendicitis with serositis	0 (0%)	1 (0.75%)	0 (0%)	1
	Acute gangrenous appendicitis	0 (0%)	0 (0%)	1 (4.35%)	1
	Chronic appendicitis	0 (0%)	1 (0.75%)	0 (0%)	1
	Lymphoid hyperplasia	0 (0%)	2 (1.5%)	0 (0%)	0.568
	Lymphoid follicular hyperplasia	0 (0%)	0 (0%)	0 (0%)	1
	Appendix measuring 10 cm × 2 cm	0 (0%)	0 (0%)	1 (4.35%)	1
	Perforated appendicitis	0 (0%)	0 (0%)	18 (78.26%)	**<0.001**
	Perforated appendicitis with fecal impaction	0 (0%)	0 (0%)	1 (4.35%)	1
	Caseating granulomatous appendicitis with mycobacterial tuberculosis	0 (0%)	0 (0%)	1 (4.35%)	1

Data are presented as frequency (%). US, ultrasound; CT, computerized tomography.

Bold values means it is statistically significant.

Regarding histopathology in the normal appendix group, no significant pathological abnormalities were found in 31 (96.87%) patients, while 1 (3.12%) patient had a normal appendix with fecal impaction (Table 3).

Regarding histopathology in the acute appendicitis group, 129 (96.99%) patients had acute simple appendicitis, 1 (0.75%) patient had acute suppurative appendicitis with serositis, 1 (0.75%) patient had chronic appendicitis, and 2 (1.5%) patients had lymphoid hyperplasia. An appendix measuring 10 cm × 2 cm, acute gangrenous appendicitis, perforated appendicitis with fecal impaction, and caseating granulomatous appendicitis with mycobacterial tuberculosis were not present in any patients (Table 3).

Regarding histopathology in complicated appendicitis, 1 (1.45%) patient had acute simple appendicitis, 1 (1.45%) patient had acute gangrenous appendicitis, 1 (1.45%) patient had an appendix measuring 10 cm × 2 cm, 18 (78.26%) patients had perforated appendicitis, and 1 (1.45%) patient had perforated appendicitis with fecal impaction. A normal appendix with fecal impaction, acute suppurative appendicitis with serositis, chronic appendicitis, lymphoid hyperplasia, and lymphoid follicular hyperplasia were not present in any patients (Table 3).

Simple appendicitis was significantly more common in the acute appendicitis group than in the complicated appendicitis group, while perforated appendicitis was significantly less common in the acute appendicitis group than in the complicated appendicitis group (*P* < 0.001) (Table 3).

In multivariate regression, WBC count and NLR were associated with AA [OR (95% CI): 1.251 (1.088–1.44) and 2.171 (1.584–2.976), respectively; *P* < 0.001], whereas neutrophil was not. NLR was associated with complicated appendicitis [OR (95% CI): 1.098 (1.02–1.181); *P* = 0.012], whereas WBC and neutrophil counts were not (Table 4).

**Table 4:** Logistic regression of variant markers for prediction of acute and complicated appendicitis.

	Multivariate		
	Odds ratio	95% CI	*P*
Acute appendicitis			
WBC (×10^3^/µL)	1.251	1.088–1.44	**0**.**001**
Neutrophil (×10^3^/µL)	1.078	0.917–1.266	0.359
Neutrophil-to-lymphocyte ratio	2.171	1.584–2.976	**<0**.**001**
Complicated appendicitis			
WBC (×10^3^/µL)	1.123	0.928 −1.357	0.231
Neutrophil (×10^3^/µL)	1.012	0.816–1.255	0.91
NLR	1.098	1.02–1.181	**0**.**012**

CI, confidence interval; NLR, neutrophil-to-lymphocyte ratio. Bold values indicate statistical significance (*P* ≤ 0.05).

**Figure 1:**
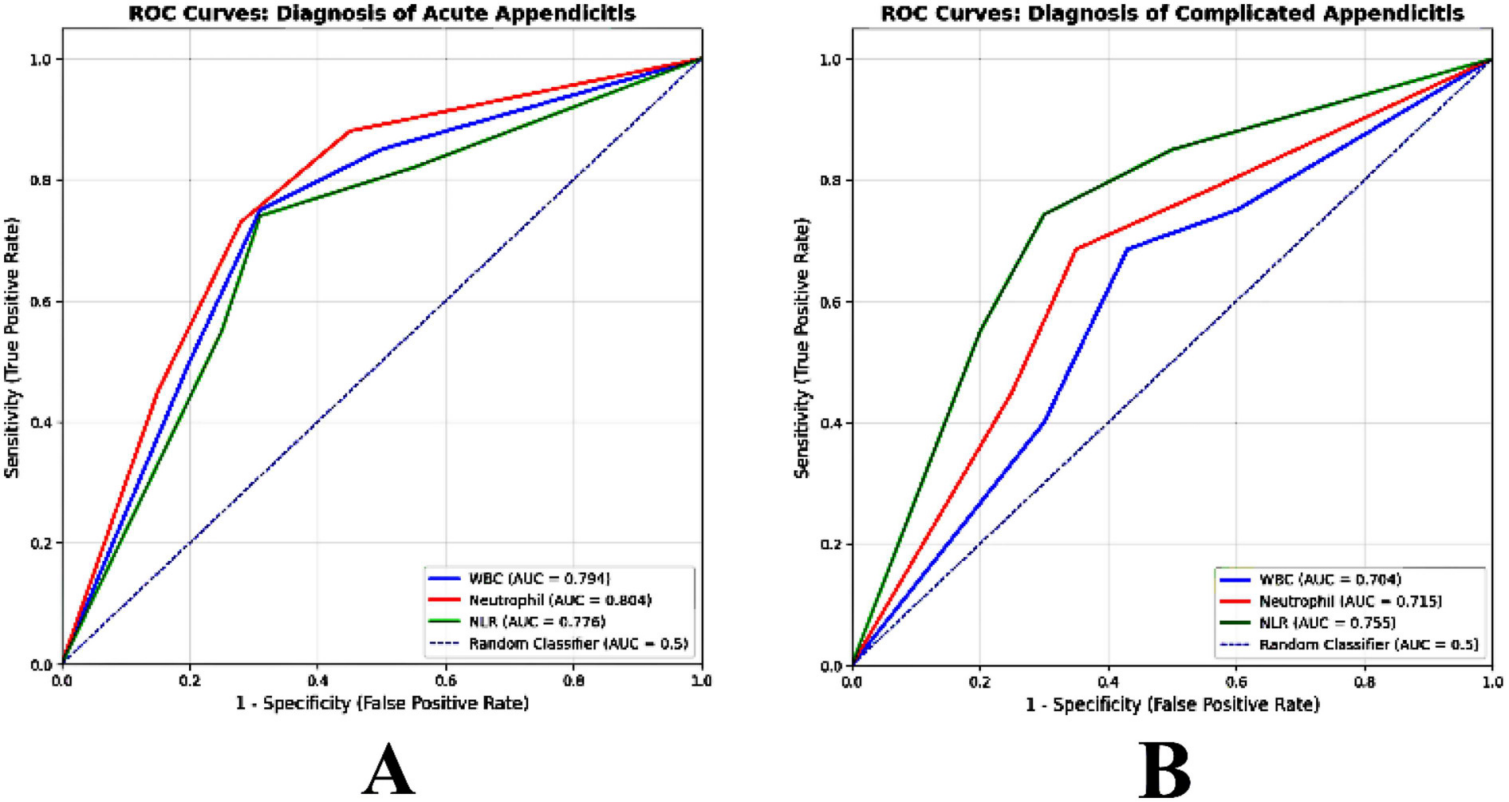
Variant markers (WBC, neutrophil, and NLR) for diagnostic performance **(A)** ROC curves for discriminating acute appendicitis (Group 2, *n* = 133) from normal appendix (Group 1, *n* = 32) and **(B)** ROC curves for discriminating complicated appendicitis (Group 3, *n* = 35) from uncomplicated acute appendicitis (Group 2, *n* = 133).

WBC count was significantly associated with acute appendicitis (*P* < 0.001 and AUC = 0.794) at a cutoff of >9.2 with 75% sensitivity, 69% specificity, 82.9% positive predictive value (PPV), and 58% negative predictive value (NPV). Neutrophil count was significantly associated with acute appendicitis (*P* < 0.001 and AUC = 0.804) at a cutoff of >6.2 with 73% sensitivity, 72% specificity, 83.9% PPV, and 57.1% NPV. NLR was significantly associated with acute appendicitis (*P* < 0.001 and AUC = 0.776) at a cutoff >1.7 with 74% sensitivity, 69% specificity, 82.7% PPV, and 57% NPV (Figure 1A).

WBC count was significantly associated with complicated appendicitis (*P* < 0.001 and AUC = 0.704) at a cutoff of >10.1 with 68.57% sensitivity, 56.98% specificity, 17.4% PPV, and 93.2% NPV. Neutrophil was significantly associated with complicated appendicitis (*P* < 0.001 and AUC = 0.715) at a cutoff of >7.8 with 68.57% sensitivity, 64.91% specificity, 20.5% PPV, and 94%NPV. NLR was significantly associated with complicated appendicitis (*P* < 0.001 and AUC = 0.755) at a cutoff of >3.5 with 74.29% sensitivity, 69.81% specificity, 24.5% PPV, and 95.4% NPV (Figure 1B).

## Discussion

AA is a condition characterized by appendix inflammation, typically caused by a blockage in the appendix, leading to an infection (9). The elevated WBC levels in AA can be explained by the body's natural immune response to the infection (10). Neutrophils are the first type of WBCs to respond to an infection and are responsible for killing and digesting bacteria. Neutrophilia is a common indicator of an acute bacterial infection, such as appendicitis (11).

A higher NLR is associated with a more severe inflammatory response (12), and studies have shown that an elevated NLR is a useful marker for the diagnosis and prognosis of AA (13–15).

To date, no study has been conducted in Jeddah to examine the relationship between these biomarkers and AA. Thus, we conducted the first study to evaluate the diagnostic value of NLR in AA in the pediatric population in Jeddah.

WBC count, neutrophil count, and NLR were found to be significantly elevated in both acute appendicitis and complicated appendicitis groups compared with those in the normal appendix group. This finding is consistent with previous studies that have demonstrated the utility of these inflammatory markers in diagnosing AA (16, 17).

The study also found that the normal appendix group had significantly higher sodium levels compared with both appendicitis groups. This observation aligns with Lindestam et al. (18) who suggested that hyponatremia may be associated with AA.

In the present study, WBC count was significantly associated with AA (AUC = 0.794) at a cutoff of >9.2, with 75% sensitivity and 69% specificity, and complicated appendicitis (AUC = 0.704) at a cutoff of >10.1, with 68.57% sensitivity and 56.98% specificity.

Prasetya et al. (19) agreed with our findings and noticed that WBC count was associated with complicated appendicitis in children (AUC = 0.644) at a cutoff of 13.63 with 66.1% sensitivity and 62.5% specificity. However, they reported that WBC count was also associated with AA. This difference may be attributed to different cutoff points. In line with our results, Eun et al. (20) reported that WBC count was significantly associated with AA in pediatric patients with 79% sensitivity and 68% specificity.

In this study, neutrophil count was significantly associated with AA (AUC = 0.804) at a cutoff of >6.2, with 73% sensitivity and 72% specificity, and complicated appendicitis (AUC = 0.715) at a cutoff of >7.8, with 68.57% sensitivity and 64.91% specificity.

In agreement with our findings, a meta-analysis by Eun et al. (20) reported that the absolute neutrophil count was significantly associated with AA in pediatric patients with 75% sensitivity and 78% specificity. In addition, Prasetya et al. (19) demonstrated that neutrophil count was significantly associated with AA in children (AUC = 0.756) at a cutoff of 64.2, with 83.1% sensitivity and 59.2% specificity, and complicated appendicitis (AUC = 0.762) at a cutoff of 80.05, with 74.5% sensitivity and 66.7% specificity. Similarly, Beecher et al. (17) reported that neutrophil count could distinguish between complicated appendicitis and uncomplicated appendicitis (AUC = 0.79, *P* < 0.001).

Our results showed that NLR was significantly associated with AA (AUC = 0.776) at a cutoff of >1.7, with 74% sensitivity and 69% specificity, and complicated appendicitis (AUC = 0.755) at a cutoff of >3.5, with 74.29% sensitivity and 69.81% specificity.

In agreement with our findings, Khan et al. (21) found that NLR was significantly associated with AA (AUC = 0.906) at a cutoff of 2.49 with 71.4% sensitivity and 12.5% specificity. In addition, Eun et al. (20) assessed NLR diagnostic utility for AA in pediatric patients. They reported that NLR was significantly associated with AA with 82% sensitivity and 76% specificity. Prasetya et al. (19) agreed with our findings and noticed that NLR was significantly associated with AA at a cutoff of 2.87, with 83.5% sensitivity and 57.7 specificity, and complicated appendicitis at a cutoff of 6.59, with 84.6% sensitivity and 56.5%% specificity. Additionally, Hajibandeh et al. (8) reported that NLR was significantly associated with AA at a cutoff of >4.7 and complicated appendicitis (AUC = 0.91) at a cutoff of 8.8 with 76.92% sensitivity and 100% specificity.

Our results were also in agreement with Bahadır et al. (22) who found that the median lymphocyte-monocyte ratio (LMR) level was significantly lower in the non-complicated and complicated appendicitis groups than in Group 1 (*P* = 0.000). The cutoff value of LMR on admission to show an association with non-complicated appendicitis was 2.98 with a sensitivity of 94% and a specificity of 96% (AUC, 0.960, *P* = 0.000). The cutoff value of LMR on admission to show an association with complicated appendicitis was 2.15 with a sensitivity of 96% and a specificity of 72% (AUC, 0.865, *P* = 0.000).

### Justification of cutoff points

The cutoff values used in this study were selected based on the cutoffs that were identified from the ROC curves of our specific dataset. The thresholds were chosen to maximize clinical utility by ensuring that clinically significant data were not overlooked, while avoiding excessive false-positive results that could lead to unnecessary investigations or interventions. Sensitivity was prioritized when early detection of acute appendicitis had a direct impact on perioperative risk stratification and surgical planning. Conversely, specificity was emphasized when the aim was to avoid overestimating associations in cases of minor or clinically insignificant findings. Thus, the chosen cutoff points represent a balance between sensitivity and specificity that was most appropriate for the clinical context of patients with acute appendicitis. The risk of threshold overfitting and the need for external validation in future prospective studies before any clinical application can be considered (23).

### Comparison of our NLR findings with other hematological indices

Our findings confirm that an elevated NLR is a valuable and readily available biomarker in supporting the diagnosis of acute appendicitis. This aligns with the established understanding of appendicitis as an inflammatory process, where neutrophilia indicates acute inflammation and lymphocytopenia may reflect systemic stress (24).

When contextualized with other hematological indices, the NLR in our study demonstrated superior diagnostic performance compared with the platelet–lymphocyte ratio (PLR). This can be pathophysiologically explained by the central role of neutrophils in the early innate immune response to appendiceal obstruction and infection. While platelets can be elevated in inflammatory states, their count is influenced by numerous other factors, making PLR a less specific marker for acute appendicitis. Our results are consistent with the meta-analysis by Yang et al. (25), who concluded that NLR had a significantly higher diagnostic accuracy than PLR for distinguishing appendicitis from other abdominal pains.

Compared to the systemic immune-inflammation index (SII), which integrates neutrophil, platelet, and lymphocyte counts (SII = platelets × neutrophils / lymphocytes), our results showed that NLR retained a comparable, if not slightly superior, diagnostic profile. The SII, designed as a more comprehensive marker of host immune and inflammatory status, has shown promise in oncological prognostication. However, in the acute setting of appendicitis, the added complexity of platelet count may not provide a significant diagnostic advantage over the simpler NLR. This observation is supported by a study by Tantawy et al. (26), who found that while both NLR and SII were significantly higher in appendicitis patients, NLR had a higher AUC for diagnosis than SII. Similarly, Ishizuka et al. (27) reported that NLR was a more sensitive predictor of complicated appendicitis than PLR.

In conclusion, while PLR and SII serve as useful inflammatory indices, the NLR appears to be the most robust and directly relevant hematological ratio for the diagnosis of acute appendicitis. Its components directly mirror the acute inflammatory and stress response characteristic of the disease. The simplicity, cost-effectiveness, and wide availability of the NLR make it an excellent adjunctive tool to clinical judgment and radiological findings in the diagnostic workup of suspected appendicitis.

### Limitations

The findings of this study are limited by the study's retrospective nature, small sample size, and single-center location. Conducting larger, multicenter, prospective studies to validate the findings and reduce the potential biases associated with retrospective data collection is recommended. The integration of NLR, along with WBC and neutrophil count, into clinical decision-making processes is recommended to improve the accuracy and timeliness of AA diagnosis in the pediatric population.

### Potential biases

As with all retrospective studies, several forms of bias must be considered:

1. Selection bias: Since only patients with both surgical and clinical documentation were included, children without complete records or without documentation may have been systematically excluded.
2. Information bias: The accuracy of data relied on the quality of medical records. Variability in documentation between clinicians, changes in diagnostic protocols over time, or incomplete recording may have influenced case classification.
3. Referral bias: As this study was conducted at a tertiary referral center, patients with more complex presentations may have been overrepresented compared with the general population of simple appendicitis patients.
4. Temporal bias: Over the study period, advances in diagnostic imaging and perioperative care may have affected detection rates and outcomes.
5. Confounding: Certain factors (e.g., associated syndromes and genetic conditions) may confound the observed data, and these variables may not have been uniformly available in retrospective records.

## Conclusion

NLR is a potentially useful adjunctive and cost-effective diagnostic marker for AA and complicated appendicitis in the pediatric population.

## Data Availability

The raw data supporting the conclusions of this article will be made available by the authors, without undue reservation.
